# Physiological and behavioural responses of wandering albatross chicks (*Diomedea exulans*) to novel and non-novel predators

**DOI:** 10.1007/s00359-026-01793-6

**Published:** 2026-01-29

**Authors:** Anais Cotton, Christophe Barbraud, Sarah Leclaire, Karine Delord, Aymeric Bodin, Antoine Stier, Cécile Ribout, Charline Parenteau, Jean-Baptiste Ferdy, Charlotte Bourgoin, Joël White, Frédéric Angelier, Pierrick Blanchard

**Affiliations:** 1 Centre de Recherche sur la Biodiversité et l’Environnement (CRBE), UMR 5300, Université de Toulouse, Toulouse INP, CNRS, IRD, Toulouse, France; 2 Centre d’Études Biologiques de Chizé, CNRS – La Rochelle Université, UMR 7372, 79360 Villiers-en-Bois, France; 3 Réserve Naturelle Nationale des Terres Australes Françaises, TAAF, rue Gabriel Dejean, 97458 Saint-Pierre, France; 4 Université de Strasbourg, CNRS, IPHC UMR 7178, 67000 Strasbourg, France; 5 Department of Biology, University of Turku, Turku, Finland; 6 Ecole Nationale Supérieure de Formation de l’Enseignement Agricole (ENSFEA), 31326 Castanet-Tolosan, France

**Keywords:** Eco-physiology, Feral cats, Landscape of fear, Predator-prey relationships, Predation risk, Predator novelty

## Abstract

In long-standing predator-prey systems, prey typically evolve costly responses to predation risk. How prey respond to novel predators is less investigated. We explored physiological (corticosterone, triglyceride), morphological (body condition) and behavioural (defensive posture) responses of wandering albatross (*Diomedea exulans*) chicks to a novel predator, the feral cat (*Felis catus*) in Kerguelen archipelago. We implemented a semi-experimental design to increase the variance in cat abundance by regulating cat populations in certain zones of the study colony. The aforementioned chick traits were then monitored and analysed at the zone scale, by comparing nests located within regulated and non-regulated zones, and at the nest scale, by recording cat abundance through intensive camera traps monitoring. This fine-scale approach further enabled us to investigate how chicks responded to southern giant petrels (*Macronectes giganteus*), an opportunistic predator-scavenger that has co-evolved with albatrosses. Cat abundances had no effect on chick traits. In contrast, higher abundances of giant petrel were associated with an elevated rate of corticosterone increase and lower triglyceride levels. In accordance with these results suggesting a more accurate perception of predation risk mediated by giant petrels than by cats, chicks were more prone to display a defensive posture when facing a giant petrel than a cat. We discuss these results in the light of contrasting evolutionary histories and predation patterns between albatrosses and their predators, and we emphasise that studies of predator impacts on prey populations must consider both the direct and indirect effects of all predators, as well as their interactions.

## Introduction

Predators influence prey population dynamics through both direct mortality and indirect effects associated with perceived predation risk, i.e. “risk effects” (Lima 1998; Creel and Christianson 2008). The latter occur when prey alter their physiology, morphology or behaviour in response to changing predation risk and that these alterations are associated with reduced performance (e.g., in reproduction or survival) (Creel and Christianson 2008). In some systems, risk effects can outweigh the impact of direct predation on prey population dynamics (Preisser et al. 2005; Zanette et al. 2024).

The predation stress hypothesis posits that predation risk induces a physiological stress response in vertebrate prey, mediated by the activation of multiple stress-coping mechanisms, including the hypothalamic-pituitary-adrenal (HPA) axis (Clinchy et al. 2013; Sheriff and Thaler 2014). The associated activation of the HPA axis results in the release of glucocorticoid (GC) hormones that triggers the activation of an emergency life-history stage (Wingfield et al. 1998) aiming to redirect resources towards immediate survival (Wingfield 2003; Wingfield and Sapolsky 2003; Angelier and Wingfield 2013). Notably, the release of GC is known to heighten vigilance and energy mobilisation in order to reduce the mortality risk of a predator attack, and thus to enhance short-term survival (Sapolsky et al. 2000; Clinchy et al. 2004, 2013; Romero 2004; Boonstra 2013).

Although it promotes short-term benefits, prolonged activation of the HPA axis is also known to incur significant costs (Sapolsky et al. 2000; Schoech et al. 2011). Numerous studies have indeed experimentally shown that the maintenance of elevated GC level over an extended period is associated with physiological (e.g., Loiseau et al. 2008; Stier et al. 2009) and cognitive (Kitaysky et al. 2003) alterations and with reduced performance in terms of growth (Wada and Breuner 2008; Grace et al. 2017), reproduction (Kitaysky et al. 2001; Angelier et al. 2009; Vitousek et al. 2014; Nelson et al. 2015), and long-term survival (Goutte et al. 2010). GC-mediated behavioural adjustments can also translate into significant costs for prey individuals (Creel and Christianson 2008). For instance, increased vigilance can reduce time spent on essential fitness-related activities, such as foraging (e.g. Brown and Kotler 2004; Caro 2005; Voellmy et al. 2014).

In changing environments, prey may be exposed to novel predators, adding complexity to the relationship between actual predation risk, physiological and behavioural responses, and ultimately, individual fitness (Sih et al. 2011; Angelier and Wingfield 2013; Crane et al. 2024). For instance, following sea ice reduction which limits their access to traditional marine prey, polar bears (*Ursus maritimus*) have only recently increasingly preyed upon common eider (*Somateria mollissima*) nests in the Arctic (Iverson et al. 2014). Consequently, while eiders exhibit heightened heart rate responses to established predators like Arctic foxes (*Vulpes lagopus*), their physiological responses to polar bears are comparatively muted, suggesting that polar bears are not yet perceived as an immediate threat (Geldart et al. 2023). Such situations, leading to a mismatch between the landscape of fear (Laundré et al. 2010) experienced by the prey and the actual landscape of risk, deserve increased attention to draw a more complete picture of predator-prey relationships, especially in the context of global change, and in particular the increase in alien invasive species.

Here, we investigated whether and how wandering albatross (*Diomedea exulans*) chicks reacted to feral cat (*Felis catus*) abundance and behaviour in Kerguelen archipelago. While cats were introduced in the 1950s in this subantarctic archipelago (Derenne 1976), the first predation events on wandering albatrosses were only evidenced in 2014 (Barbraud et al. 2021) despite longstanding and extensive albatross monitoring, emphasising that feral cat has represented a threat on albatross chicks only very recently in this system. Risk effects are expected in wandering albatross chicks because predation threats may not only trigger a state of chronic stress (i.e. stress-mediated costs) (Cyr and Romero 2007; Scheuerlein et al. 2001; Ibanez-Alamo et al. 2011; Dulude-de Broin et al. 2020; Mohring et al. 2023), but also anti-predator behaviours, such as food regurgitation or decreased resting periods and increased vigilance bouts, as shown previously in procellariform chicks, including wandering albatrosses chicks in Kerguelen (Barbraud et al. 2021; Blanchard et al. 2024). Therefore, risk perception may translate into an altered nutritional status that can be assessed through metabolic and hormonal level (increased baseline glucocorticoids level, Cherel et al. 1988; Lynn et al. 2001; Angelier et al. 2015; Krause et al. 2016; and lower triglyceride level, Guglielmo et al. 2002; Fokidis et al. 2012; Eby et al. 2023). We thus hypothesized that chicks experiencing a higher level of fear should bear the costs of chronic stress (Kitaysky et al. 2003) and thus display altered corticosterone profiles, corticosterone being the main glucocorticoid in birds. More precisely, if chicks perceive cats as threats, we expected increased cat abundance to trigger higher baseline or stress-induced corticosterone levels (Schoech et al. 2011). In addition, chicks exposed to increased cat abundance should have a lower body condition, lower triglyceride level and higher baseline corticosterone level as a result of an altered nutritional status (food-mediated costs of predation risk, Creel 2018).

We investigated risk effects mediated by feral cats on albatross chicks at two spatial scales. Firstly, we used a cat regulation protocol to experimentally reduce feral cat abundance in some parts of the study colony (regulated zones) but not in others (non-regulated control zones) (Blanchard et al. 2024) and compared chick physiological parameters and body condition between both treatments. Secondly, we focused on the nest scale through camera trap monitoring and investigated chick response to varying levels of cat abundance, benefiting from the experimentally increased variance in cat abundance. Here, we further took advantage of the presence of the opportunistic predator-scavenger southern giant petrel (*Macronectes giganteus*) in the study area to compare chick responses to novel and former predator species. Although predation of albatross chicks by giant petrels has been firmly reported only recently in Kerguelen (Blanchard et al. 2024), both species have co-evolved in Kerguelen and the presence of giant petrels around albatross nests, sometimes harassing chicks has been reported for decades (Kidder 1876; Derenne 1976; Blanchard et al. 2024). Predation of wandering albatross chicks by giant petrels has further been reported in other subantarctic islands (Dilley et al. 2013). Finally, still at the nest scale, we considered chick behavioural responses and tested whether predator species identity, i.e. cat or giant petrel, influenced the probability for a chick to display a defensive behaviour when facing a predator, hypothesising that any change in chick physiological parameters or body condition should parallel a change in behavioural responses.

## Materials and methods

### Study area

This study was conducted from December 2021 to July 2022 in the Kerguelen archipelago, southwestern Indian Ocean. Kerguelen includes a main island (“Grande Terre”, ~ 6700 km^2^) and hundreds of smaller islands. Our study colony was localised at Cap Cotter (49.057867°S, 70.304915°E) and covers an area of ~ 20 km^2^, dominated by the native herbaceous perennial *Acaena magellanica*. In the 2021/2022 breeding season, 73 eggs were laid, from which 57 chicks hatched.

### Wandering albatross

Adults arrive at the breeding sites in November and females lay a single egg from late December to early January. Then, both parents incubate alternately for about 80 days (Weimerskirch et al. 1993). After hatching (around mid-March), partners alternate chick brooding and short foraging trips for about one month (Weimerskirch et al. 2000). The chick is then left alone, and parents mix short (up to 4 days) and long (up to 27 days) trips to regularly feed their chick until late November, when fledging occurs (Weimerskirch et al. 1993).

### Overview of the study schedule

In the 2021/2022 breeding season, chicks were monitored from hatching to the end of the study (i.e. end of July) or to their death, and were captured once a month (e.g., in April, May, June and July), as part of different research projects (e.g. Blanchard et al. 2024; Bourgoin et al. 2024). In the present paper, we excluded the brooding phase in order to avoid any parental interference with the chick’s parameters of interest (Rensel et al. 2010; Dupont et al. 2021).

For all analyses involving physiological parameters and body condition, and thus chick captures, at both the zone and the nest scales (see below), we therefore considered the period between the May capture (i.e. brooding ended for all chicks) and the June capture. Considering the July capture instead of the June capture would have further decreased our sample size due to additional chick mortalities (by nest flooding, cat / giant petrel predation and poor parental care as detailed in Blanchard et al. 2024). This led to a sample size of 27 chicks.

For the analyses involving chick behaviour monitored by camera traps (i.e. at the nest scale, see below), we considered, for all chicks individually, the period from the end of their brooding to their death (or the end of the study) in order to increase our sample size. This led to 47 chicks.

### Cat control design: cat abundance at the “zone scale”

The studied colony was divided into 4 zones of 2 km^2^, chosen in similar habitats: 2 zones regulated by feral cat hunting and 2 non-regulated zones (Blanchard et al. 2024). We performed two cat regulation operations (26 February 2022–6 March 2022 and 24 April 2022–14 May 2022) using double-door traps, leg-hold traps, and shooting with riffle Tikka T3 × 222. Overall, 30 cats were killed in regulated zones. Consequently, while there was no significant difference in cat abundance between regulated and non-regulated zones before cat control operations started (Blanchard et al. 2024), significantly more cats were observed in the two non-regulated zones as compared to the two regulated zones after cat control operations (kilometric indexes of 0.14 and 0.18 cat/km for the non-regulated zones and of 0.02 and 0.04 cat/km for the regulated zones, respectively, measured from 29 May to 10 June 2022, i.e. during the period considered in this study). More logistical and statistical details on cat control can be found in Blanchard et al. (2024).

### Camera traps: cat and giant petrel abundances at the “nest scale”

We used camera traps (Reconyx HP2X or PC 900) between the captures of May and June for all chicks to (1) assess the abundance of predators at the nest scale and, (2) record chick behaviour when facing a predator. Camera traps were programmed to take a photograph every 2 min during the entire period.

1. From May to June, camera traps were positioned in front of all of the 27 nests containing live chicks. Up to 37% of the photos could not be used because of snow, fog, or damage from reindeer (*Rangifer tarandus*) or elephant seals (*Mirounga leonina*). We decided to consider nests with at least 80% of usable photos, resulting in a final dataset of 24 nests. Because these missing photos reduce the continuity of visual coverage, it is possible that some predator encounters, particularly brief ones, were not captured. However, by applying this 80% threshold, we ensured that the retained nests still provided sufficiently reliable information on predator presence around the chicks. From these 24 nests, we individually analysed 259,592 photos to determine “giant petrel abundance” and “cat abundance” at the nest scale by dividing the total number of predators of each species identified on the photos (including flying giant petrels) by the total number of photos taken during the same period, excluding unusable photos (e.g. snow on the lens). Both cat and giant petrel average abundances varied from 0 to 0.01 individual per picture.
2. For the investigation of chick behaviour during the post-brooding stage, we individually analysed 2,783,475 photos from the 47 nests. For each photo where the predator faced the chick and the chick acknowledged the presence of the predator (i.e. looking at the predator, see Fig. 1; *n* = 263 for cats and *n* = 1594 for giant petrels, for 38 different nests), we recorded the predator species and whether or not the chick was erected, i.e. in an upright posture, with the neck raised, typically with eyes wide open and clapping bill (e.g. Dilley et al. (2013), Fig. 1 left panel). Despite this difference in total observations, the average number of observations per encounter event (i.e., all observations of the same predator within a 12-hour window) was relatively similar between predator species, with a mean of 6.70 for petrels and 6.26 for cats suggesting that the sampling effort per event was broadly comparable across predator species. Visits by the same predator species were treated as a single “event” if they occurred within 12 h of each other, and as distinct events if separated by more than 12 h. This 12-hour threshold was chosen to define separate predator encounters, accounting for individuals that may remain near a nest for several hours, sometimes outside the camera’s view, and then return. In some cases, a predator stayed in front of the camera for over six hours. Using this threshold ensures that repeated observations of the same individual are treated as a single encounter. An “event” thus corresponds to all photos in which the same predator is observed within a 12-hour window. Each observation (photo) is considered individually, and the “number of the encounter event” refers to the event identity (ranging from 1 to 286 across all nests), allowing us to account for repeated observations of the same predator and to avoid bias from which predator is seen first by a chick.
  **Fig. 1:**
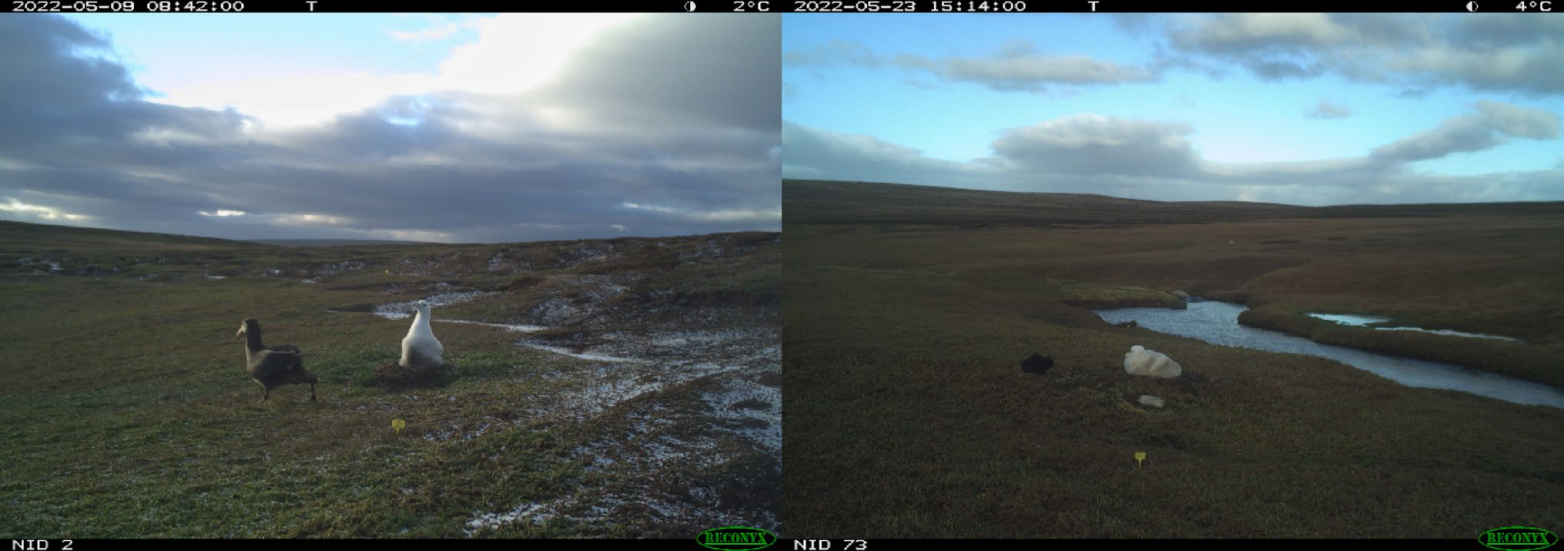
Albatross chick reaction when facing a southern giant petrel (left panel) and a cat (right panel)

### Chick’s physiological parameters and body condition: capture sessions

Chicks (*n* = 27) were captured in May (9–13 May; mean age = 57 days, range: 48–67 days) and in June (6–10 June; mean age = 84 days, range: 75–95 days). The lengths of the head (from the occipital bone to the extremity of the beak), the beak, both tarsi, and the width of the beak were measured with a calliper (± 0.1 mm) and the lengths of both wings with a ruler (± 1 mm). Finally, chicks were weighed to the nearest 50 g using a Salter scale. Chick structural size and body condition were determined as follows. We (1) conducted a Principal Component Analysis (PCA) on the standardised aforementioned measurements and used the scores from the first axis (89.4% of the total variance) as an index of structural size, with higher scores indicating larger chicks, and (2) performed a regression of chick mass against this structural size index (R² = 0.652) and used the residuals as an index of body condition (see Blanchard et al. 2007 for the same approach in the same species).

Two blood samples were collected from the tarsal vein of each chick. The first blood sample was collected within 4 min of capture (from the moment the chick spotted the biologists) and was defined as the baseline corticosterone level, as we detected no effect of the duration of the blood sample on the corticosterone hormone level (F_1,22_ = 1.053, *p* = 0.316). The second sample was collected at the end of the capture, with an average delay of 9.93 min (± 0.41 SE; range: 7–17 min) between both samples. Blood samples were centrifugated in the field and frozen for further analyses in the laboratory. Baseline corticosterone levels (ng/mL) were measured using an enzyme-linked immunosorbent assay (ELISA) kit (Demeditec, DE4164, lot 61K093), following the protocol described in Kinn Rød et al. (2017) on the first blood sample. The corticosterone level of the second sample was determined using the same method, allowing calculation of the plasma corticosterone elevation per unit time ((ng/mL)/min; hereafter “rate of corticosterone increase”) as (Corticosterone value of the second sample – Corticosterone value of the first sample) / elapsed time between the two samplings. We observed a generally linear relationship between the rate of corticosterone increase and the time of the second blood sample. However, beyond 14 min, this relationship was no longer linear, and one nest with a single measurement at 17 min was excluded from all subsequent analyses. Therefore, the rate of corticosterone increase was not significantly related to the time of the second blood sample (F_1,24_ = 1.08, p-value = 0.310), indicating that our calculation of corticosterone change per unit time is not biased by the timing of the second sampling and can be considered a reliable measure of the hormonal response. Intra-assay variability and inter-assay CVs were 7.52% and 7.49%, respectively. Triglyceride levels (mmol/L*)* were determined using the Mission^®^ Cholesterol Meter, an automatic reader (ACON laboratory in San Diego, California, US) on the first blood samples. Finally, the chick sex was obtained by using molecular techniques (PCR) as detailed in Blanchard et al. (2007). All parameters were determined for all chicks (*n* = 27), except triglyceride levels (*n* = 23) for technical reasons.

## Statistical analysis

All statistical analyses were performed in R, version 4.3.2 (R Core Team 2023). Assumptions for linear and generalised linear models (overdispersion, homoscedasticity, independence, and normality of model residuals) were checked using both graphical inspection (QQ-plots, residuals vs. fitted plots, and histograms of residuals; Zuur et al. 2009; Zuur et al. 2010). When assumptions were not met, data were log-transformed, as specified for each analysis. This approach allowed us to evaluate model assumptions while acknowledging the limitations imposed by the small sample size.

Before investigating the effects of predator abundance at both scales on chicks physiological (baseline corticosterone, rate of corticosterone increase and triglyceride levels) parameters and body condition, we tested for correlations between those parameters using Pearson correlation tests. Baseline corticosterone and triglyceride level were log-transformed to meet normality assumption.

### Cat abundance effect at the “zone scale”

In order to decrease the number of variables included in the models, we performed preliminary analyses and checked whether measured traits in June were impacted by their values in May and by chick sex and age, using linear models, t-tests or Pearson correlation tests depending on the variable type and distribution. As no relationship reached statistical significance (all p-values > 0.105), we used mixed models including only zone status (regulated or non-regulated) as an explanatory variable and zone identity as a random term. Although the rate of corticosterone increase showed a marginal, non-significant trend with sex (F_1,17_ = 4.34, p-value = 0.053), tending to be higher in males, this effect was not retained in the final models. We ran separate models for each physiological parameter and for body condition given the limited sample size. Baseline corticosterone and triglyceride level were log-transformed. As the 4 zones were not designed as adjacent (Blanchard et al. 2024), some of the 27 nests (*n* = 7) included in the study colony were localised outside the zones. We thus performed the analyses both without these nests and, in order to increase sample size, with these nests attributed to the closest zone. Samples sizes were thus of 20 and 27 chicks for all traits for both analyses respectively, except for triglyceride level (*n* = 17 and *n* = 23 respectively) and the rate of corticosterone increase (*n* = 19 and *n* = 26 respectively).

### Cat and giant petrel abundances effects at the “nest scale”

Similar to analyses performed at the zone scale, preliminary analyses revealed no significant relationships between chick sex, age and the values of chick traits in May and their values in June in this dataset neither (all p-values were > 0.010, except for one case where the baseline corticosterone value in May positively influenced the baseline corticosterone in June (F_1,22_ = 3.52, p-value = 0.074)). We thus used linear models solely including the abundance of predator at the nest scale as an explanatory variable, built separately by predator species to allow model convergence. Triglyceride level was log-transformed. Samples sizes were of 24 for all parameters, except for triglyceride level (21) and the rate of corticosterone increase (23).

### Chick reaction to encounters with predators at the “nest scale”

Finally, we conducted two complementary generalised linear mixed-effects models (GLMMs) to examine whether predator identity (cat or giant petrel) shaped the chick probability to react (erect or not) when facing the predator as a dependant variable. In the first analysis, chick age (log-transformed) was included as a fixed effect, while the number of the encounter event and nest identity were included as random terms. As an “event” could last from minutes to hours, it often generated multiple photos: a brief encounter could produce only one photo with no response, whereas a prolonged or repeated encounter could produce many photos and a behavioural response. To account for potential sequential effects within events, the second GLMM was conducted including only the first photo of each event, with chick age (log-transformed) as a fixed effect and nest identity as a random term.

## Results

Baseline corticosterone was positively correlated with the rate of corticosterone increase (*r* = 0.47, df = 24, p-value = 0.016) and negatively correlated with triglyceride level (*r* = -0.47, df = 21, p-value = 0.025) and chick body condition (*r* = -0.64, df = 25, p-value < 0.001). The rate of corticosterone increase was also negatively correlated with triglyceride level (*r* = -0.52, df = 20, p-value = 0.014). Body condition was marginally positively correlated with triglyceride level (*r* = 0.41, df = 21, p-value = 0.052). No significant correlation was found between the rate of corticosterone increase and body condition (*r* = -0.09, df = 24, p-value = 0.656).

### Cat abundance effect at the “zone scale”

**Fig. 2:**
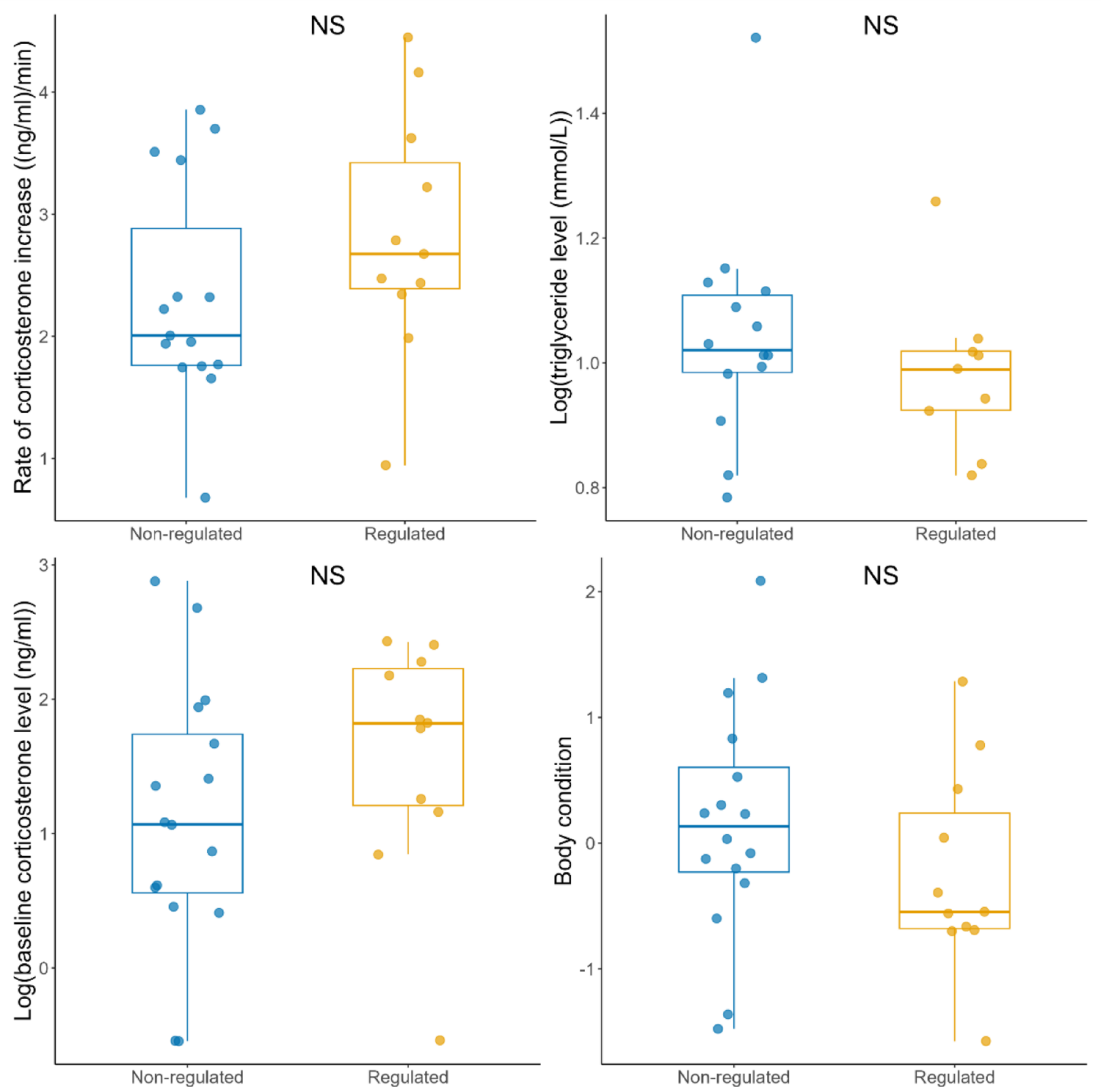
Effect of zone type (regulated - i.e. cats hunted - and non-regulated - i.e. cats non-hunted) on chick physiological parameters and body condition in Kerguelen. No significant effects were detected, even when including nests located near but outside the zones (F < 1.34, all p-values > 0.258)

None of the measured chick physiological parameters nor body condition showed any significant differences between regulated (i.e. cats being hunted) and non-regulated zones (F < 1.33, all p-values > 0.263), even when increasing the sample size by attributing nests outside the zones to the closest zone (F < 1.34, all p-values > 0.258, Fig. 2).

### Cat and giant petrel abundances effects at the “nest scale”

**Fig. 3:**
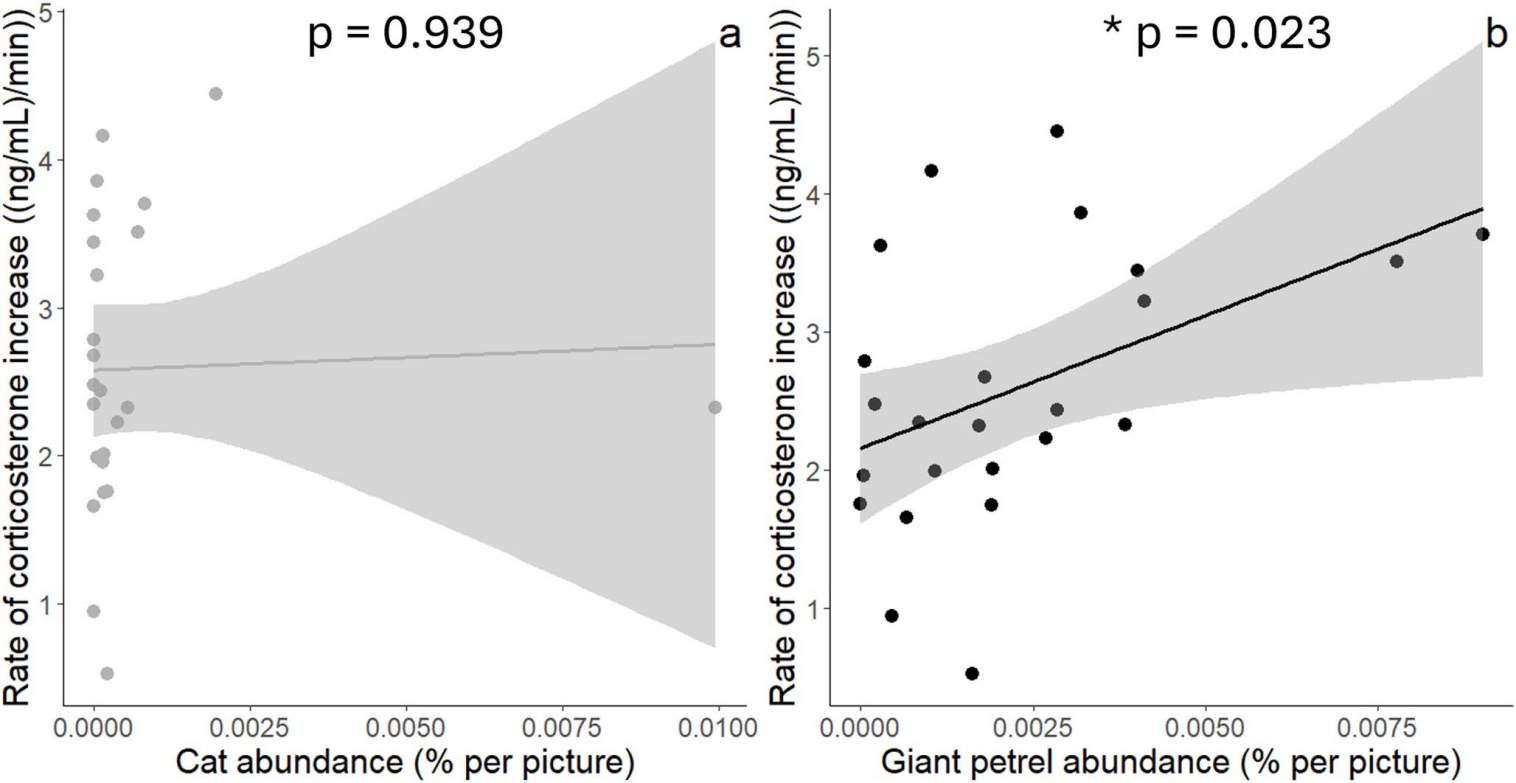
Rate of corticosterone increase ((ng/ml)/min) in wandering albatross chicks in Kerguelen, in relation to cat (**a**) and giant petrel (**b**) abundances (average number of individuals per picture) (Cat: F_1,21_ = 0.01, p-value = 0.939, R² = 0.01, Cohen’s f² = 0.01; Giant petrel: F_1,21_ = 5.98, p-value = 0.023, R² = 0.22, Cohen’s f² = 0.29). Cook’s distance was calculated, and no statistical outliers were detected

**Fig. 4:**
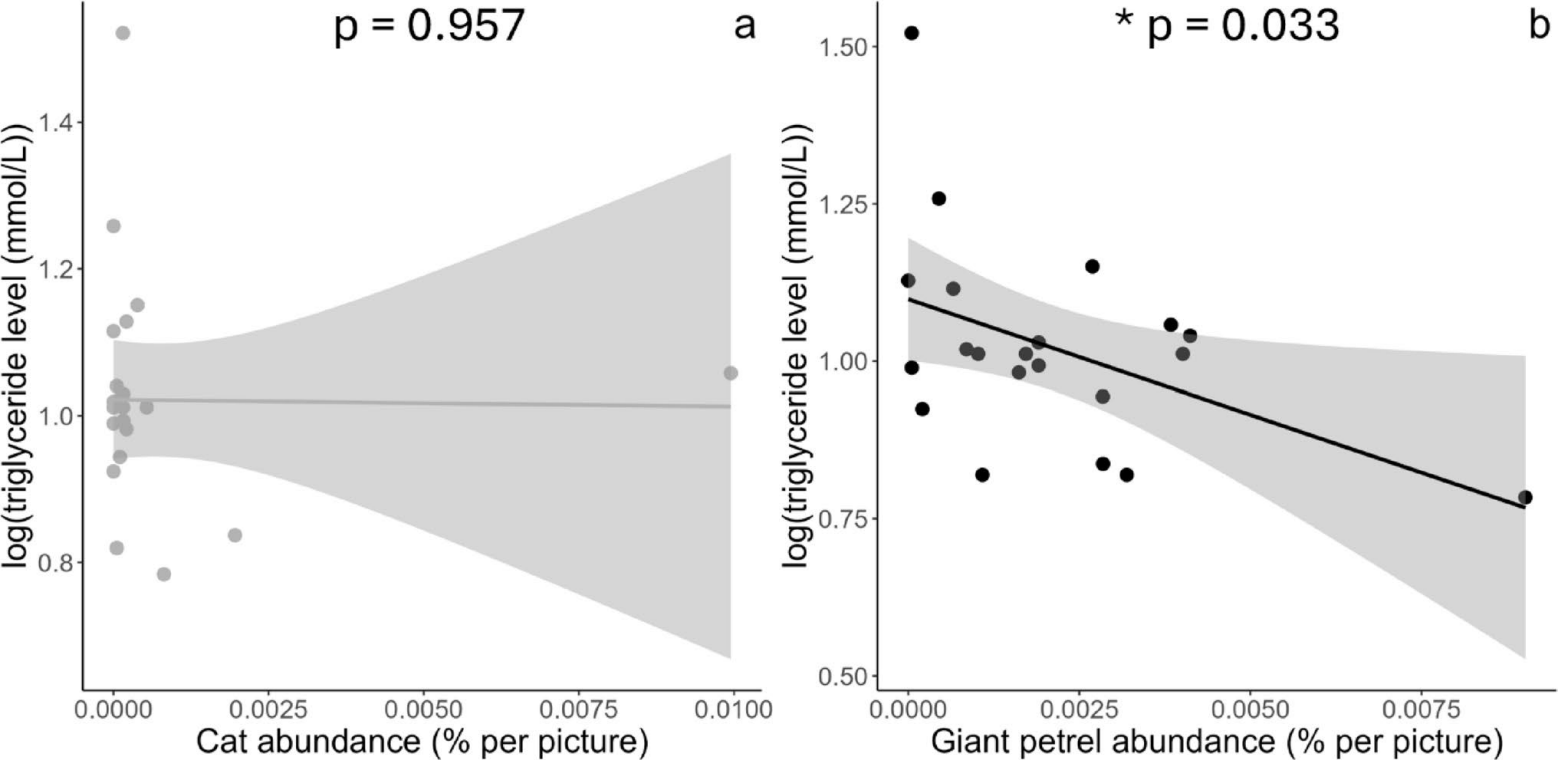
Triglyceride level (mmol/L; log transformed) in wandering albatross chicks in Kerguelen, in relation to cat (**a**) and giant petrel (**b**) abundances (average number of individuals per picture) (Cat: F_1,19_ = 0.01, p-value = 0.957, R² = 0.01, Cohen’s f² = 0.01; Giant petrel: F_1,19_ = 5.29, p-value = 0.033, R² = 0.22, Cohen’s f² = 0.28). Cook’s distance was calculated, and no statistical outliers were detected

Chicks physiological parameters and body condition did not relate to cat abundance (all F < 0.67, all p-values > 0.422; Figs. 3a and 4a), when considering all data points. Higher giant petrel abundance was significantly associated with both higher rate of corticosterone increase (F_1,21_ = 5.98, p-value = 0.023, R² = 0.22, Cohen’s f² = 0.29) (Fig. 3b) and lower triglyceride level (F_1,19_ = 5.29, p-value = 0.033, R² = 0.22, Cohen’s f² = 0.28) (Fig. 4b). When petrel abundance increased twofold, the rate of corticosterone increase raised by approximately 146%, while triglyceride level (log-transformed) decreased by about 16%. Neither chick body condition nor baseline corticosterone related to giant petrel abundance (Condition: F_1,22_ = 3.54, p-value = 0.469, R² = 0.02, Cohen’s f² = 0.02; Baseline corticosterone: F_1,22_ = 2.07, p-value = 0.164, R² = 0.09, Cohen’s f² = 0.09).

### Chick reaction to encounters with predators at the “nest scale”

**Fig. 5:**
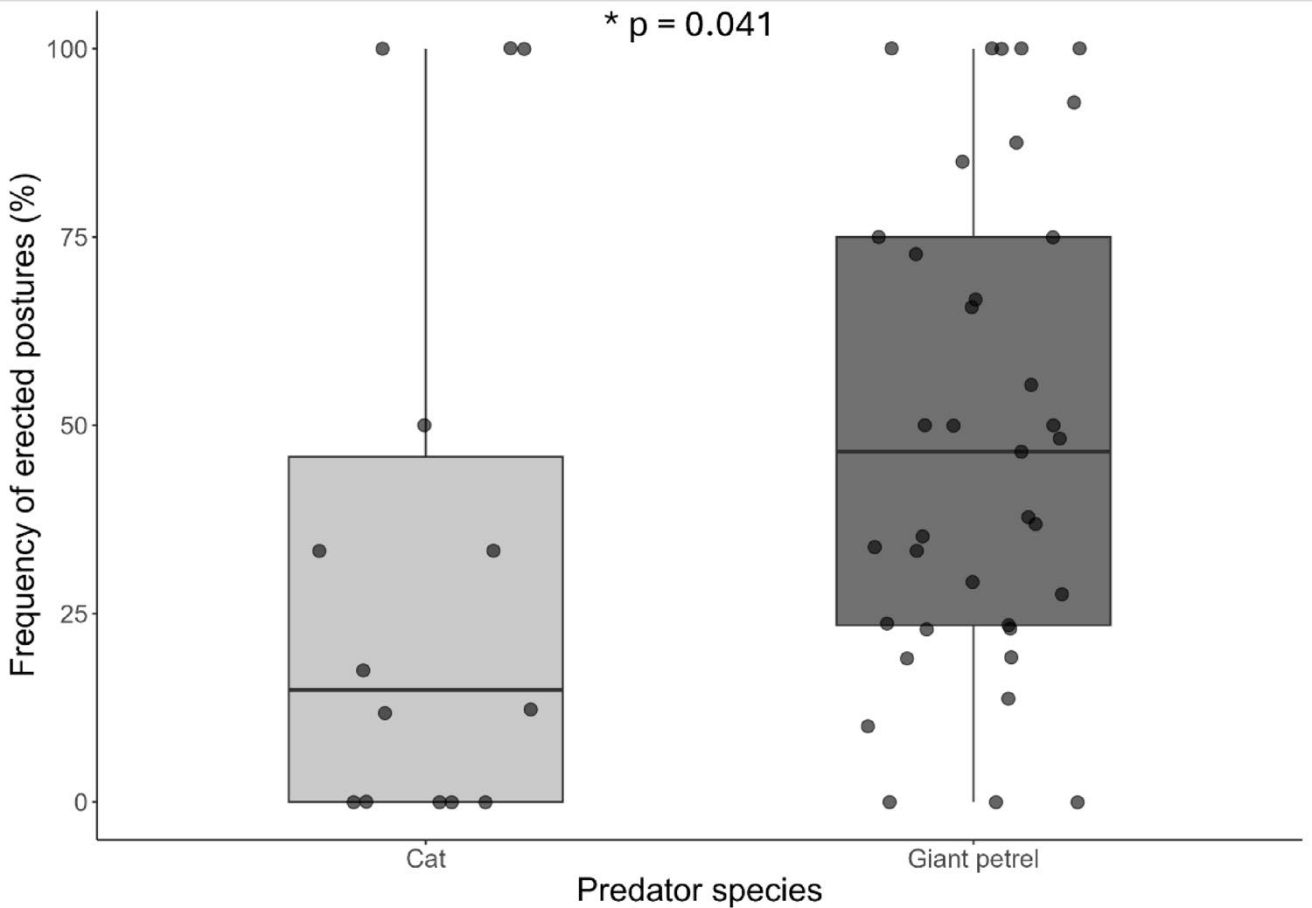
Frequency of erected postures (% per nest, *n* = 38 nests, totalling 1594 encounters with giant petrels and 263 with cats) in wandering albatross chicks facing a predator in Kerguelen, in relation to predator species (χ²₁ = 4.18, df = 1, p-value = 0.041)

Chicks erected in 36% of encounters with a giant petrel, compared to 16% of encounters with a cat (Fig. 5; see also Fig. 1). This difference was statistically significant (χ²₁ = 4.18, df = 1, p-value = 0.041, OR = 2.79, 95% CI: 1.04–7.44). Chick age had no effect on the probability of reacting to an approaching predator (χ²₁ = 0.21, df = 1, p-value = 0.643, OR = 0.79). When considering only the first photo of each event, the difference became even more pronounced (χ²₁ = 5.07, df = 1, p-value = 0.024, OR = 2.57, 95% CI: 1.13–5.84).

## Discussion

While the number of studies investigating how the landscape of fear shapes predator-prey relationships sharply increased in the last decades, those targeting risk effects mediated by a novel predator species are less common, especially those including physiological aspects (but see e.g. Geldart et al. 2023). Here, we investigated whether and how feral cat abundance and behaviour drove physiological, morphological and behavioural traits in a novel prey, wandering albatross chicks in the Kerguelen archipelago. We set up a semi experimental design to reduce feral cat abundance in some parts of the study colony. In addition, we took advantage of the presence of the opportunistic predator-scavenger southern giant petrel in the study area to test whether prey response could depend on predator species identity. Although our sample sizes are modest (27 chicks for physiological analyses and 38 chicks for behavioural analyses), which may reduce statistical power, the combination of these results suggests that the presence of cats has no major effect on our physiological and behavioural variables of interest in wandering albatross chicks. Overall, it suggests that chicks weakly perceive predation risk mediated by cats in this population, although further studies with larger sample sizes are warranted to generalise this interpretation and to confirm these findings. On the contrary, chicks exposed to higher petrel abundance at the nest scale showed a clear response, with higher rate of corticosterone increase and reduced triglyceride level. Finally, chicks showed a higher probability to display an erect posture when facing an approaching giant petrel than a cat, supporting further the idea of an increased perception of risk mediated by giant petrels than by cats.

### Responses to feral cats

Our data indicate that there are no differences in physiological parameters or body condition between chicks in regulated and non-regulated zones. We also did not report any major relationship between feral cat abundance and chick physiological parameters or body condition at the nest scale. Although our sample sizes are modest, the combination of these results suggest that the presence of cats has no major effect on wandering albatross chicks, i.e. that chicks weakly perceive predation risk mediated by cats in this population. This interpretation is further supported by chicks being rarely erected when facing a cat in our study (i.e. 2.25 less erected postures than when facing a giant petrel; Fig. 5). Hence, an increased abundance of feral cats is not associated with a drastic corticosterone stress response, nor with increased vigilance and defensive behaviour (e.g., regurgitation of stomach oil). Because these three variables are associated with increased metabolic costs (see below), it also probably means that the presence of cats does not entail any important energetic costs to wandering albatross chicks. Wandering albatrosses may not perceive feral cats as a threat, as predation is recent (Barbraud et al. 2021; Blanchard et al. 2024), which may explain their relative naiveté (sensu Cox and Lima 2006) when facing cats. Moreover, the cat–albatross system is highly specific here, limiting opportunities for chicks to develop risk perception mediated by cats: although the overall cat impact on albatross population is strong (population modelling showed that the albatross population would decline by 2.7–4.5% per year without cat control in Kerguelen, Barbraud et al. 2021), probably only a small fraction of the cat population attack chicks (Blanchard et al. 2024), and many attacked chicks die from predation or from their wounds in the following days (Barbraud et al. 2021; Blanchard et al. 2024). Hence, chicks seldom have the opportunity to learn to fear cats (Preisser et al. 2005; Crane et al. 2024), as non-successful predation events might be relatively rare. While antipredator behaviour can in theory develop through individual learning or natural selection when encounters with a novel predator are recurrent, in this population these mechanisms are likely limited. Social transmission of fear is also unlikely, as cat attacks mostly occur when parents are absent, preventing vertical transfer of fear from adults to chicks (Barbraud et al. 2021; Blanchard et al. 2024). Over longer timescales, persistent predation pressure could favour individuals that respond more effectively to cats, potentially leading to the emergence of stronger antipredator behaviours through selection. However, given the long generation time of wandering albatrosses and the relative rarity of encounters, such learned or selected fear is expected to remain limited (Caro 2005). Non-agonistic encounters, such as cats scavenging at nests, are unlikely to provide strong cues for learning. The above speculations have also been proposed as a hypothesis to explain why brooding duration was independent of feral cat abundance in this population (Bourgoin et al. 2024), despite longer brooding being expected to reduce predation risk (Catry et al. 2009).

Alternatively, because the chicks were not sampled immediately after an encounter with a cat, the physiological parameters we considered may be too labile to reflect the physiological costs of an increased predation risk perception mediated by cats, particularly if corticosterone levels rapidly return to baseline once the stressor has ceased (Zimmer et al. 2019; Vitousek et al. 2019). This temporal mismatch between continuous predator encounters and monthly physiological sampling could obscure short-term stress responses, especially to cats, and allows us to only detect persistent/long-term effects of cat encounter on chick physiology. In other words, chicks could physiologically respond to the presence of cats but these responses could not last long enough to be detected at the time of the sampling (especially when the sampling occurred a long time after the encounter). Indeed, previous studies have found that heart rates return to baseline within a few hours after a stressor occurs in wandering albatross (Weimerskirch et al. 2002) and, in another procellariform species, the snow petrel (*Pagodroma nivea*), data showed that corticosterone level return to pre-stressor level quite rapidly too (i.e. ~20 min, Angelier et al. 2015). However, this hypothesis is quite unlikely here because we report that these physiological variables were correlated with the presence of another predator, the giant petrel, suggesting that they can indeed be affected by long-term predation risk in wandering albatross chicks, as found in other systems (Scheuerlein et al. 2001; Cockrem and Silverin 2002; Fontaine et al. 2011; Mohring et al. 2023). Therefore, the pattern we report probably arises from a real difference in how albatross chicks perceive both predator species.

### Responses to giant petrels

Unlike for cats, a higher abundance of giant petrels at the nest scale was clearly associated with higher rate of corticosterone increase and lower triglyceride level at the timing of sampling, suggesting the existence of predation risk perception mediated by giant petrels in albatross chicks in this system. This is further supported by chicks often displaying a defensive posture when facing a giant petrel, as compared to situations where they face a cat (Fig. 5). The existence of a threat perception mediated by giant petrels is not surprising in this colony as agonistic encounters, implying harassment (authors, personal observations), between albatross chicks and giant petrels, more frequent than with cats (see also results), are also probably associated with less killing (only two reports of petrel giant predation on albatross chicks in Kerguelen overall, with 6% (*n* = 2) of mortality overall as compared to 24% (*n* = 8) for cats, in the study colony and year, Blanchard et al. 2024), providing opportunity for chicks to learn fear. Moreover, risk perception is probably well established because both species share a common evolutionary history, and agonistic interactions between giant petrels and seabirds, including wandering albatrosses (Cox 1978; Dilley et al. 2013; Barbraud et al. 2021; Blanchard et al. 2024), are commonly reported (e.g. Berruti 1979; Forster and Phillipps 2009; Risi et al. 2021; Wagner et al. 2024). Non-exclusively, the size ratio between cats and giant petrels, with giant petrels being much taller than cats (see Fig. 1), may also, in itself, explain why chicks may be more prone to display a defensive posture when facing a giant petrel.

The effects of giant petrel abundance on the rate of corticosterone increase and on triglyceride level may result from two non-exclusive mechanisms linking predation risk and the HPA axis (Clinchy et al. 2013; Sheriff and Thaler 2014). First, predation risk can activate the HPA axis, leading to the release of corticosterone by the adrenal glands (Wingfield et al. 1998; Wingfield 2013; Angelier and Wingfield 2013). In turn, this corticosterone stress response can activate metabolic processes to prepare the organism to cope with energy-demanding activities such as escaping or fighting predatory events (“the preparative hypothesis”, Romero 2002). Indeed, previous studies have reported not only that corticosterone is tightly linked to metabolism and energy expenditure (Astheimer et al. 1992; Jimeno et al. 2018; Jimeno and Verhulst 2023), but also that corticosterone level can be increased during periods of expected higher energetic demands (reviewed in Romero 2002). Corticosterone hormones may then directly affect the individual energy consumption and deplete energy reserve (Sapolsky et al. 2000; Sheriff and Thaler 2014), which may explain our observed lower triglyceride level in response to high giant petrel abundance (Remage-Healey and Romero 2001; Butler et al. 2020). Second, the effects of giant petrel abundance on the rate of corticosterone increase and on triglyceride level may result from the agonistic encounters by giant petrels which may induce behavioural changes, such as increased vigilance and activity in the nest, or energy-demanding defensive behaviours (e.g. regurgitating oil and food, which may encourage harassment) as suggested by our results and previous studies (Barbraud et al. 2021). These changes may in turn increase energy expenditure, induce nutritional stress, and thus, result in reduced growth and body condition (Creel and Christianson 2008). Yet, in our study, we did not detect a significant effect of giant petrel abundance on chick body condition at the time of measurement, suggesting that the physiological stress responses observed may not translate into immediate, easily detectable fitness costs. However, physiological stress responses such as increased corticosterone secretion and reduced triglyceride levels may still reflect elevated energetic expenditure and short-term nutritional stress that are not necessarily captured by single measures of body condition. Importantly, repeated harassment by giant petrels throughout the chick-rearing period could have cumulative effects on growth trajectories and energy allocation. In long-lived seabirds, fledging mass and body condition are strong predictors of post-fledging survival and recruitment, particularly in albatrosses, where variation in growth rate and fledging condition has been linked to juvenile survival probabilities (Weimerskirch et al. 2000). Thus, while physiological stress responses in our system do not appear to translate into immediate reductions in body condition, they may still incur delayed or cumulative fitness costs by subtly altering growth, delaying fledging, or reducing condition at fledging. Such carry-over effects may only become apparent at later life stages and would require long-term monitoring to be fully quantified. Climate-driven changes in marine ecosystems may further modify these dynamics by altering predator abundance, distribution, or foraging behaviour. Such shifts could affect encounter rates with both historical and novel predators, potentially amplifying or mitigating non-lethal stress and its consequences for chick fitness (Barbraud et al. 2012; Jenouvrier 2013). These considerations highlight the importance of long-term monitoring to understand how environmental change may shape predator–prey interactions and their long-term effects on seabird populations.

### Conclusion

Overall, these results and previous studies on this system (Barbraud et al. 2021; Blanchard et al. 2024) suggest that a small fraction of the cat population has a strong impact on albatross demography through direct effects (i.e. killing), while risk effects mediated by cats are probably rare, because of the novelty of this predator-prey system and/or specific predation patterns preventing chicks from rapidly developing risk perception mediated by cats. In contrast, a probable larger fraction of the giant petrel population may impact chick physiology through harassment (this study), while direct predation remains probably rare (Blanchard et al. 2024). Predator management for cats can be effective for the fraction of the population that preys on wandering albatross chicks, as cats are non-native and can be targeted locally (Blanchard et al. 2024). In contrast, mitigating giant petrel predation is challenging due to their mobility, protected status, and attraction to food brought to nests by parents. These constraints limit practical management options and should be considered when evaluating future conservation strategies. To which extent these physiological effects translate into fitness costs for albatross chicks, as well as how the chicks - giant petrels and the chicks - feral cats systems might interact, now and in the coming years (Blanchard et al. 2024), requires further investigation.

## Data Availability

The datasets generated and analysed during the current study are available to reviewers upon request. Following publication, all data will be deposited in an open-access repository and made publicly available.
