# Correction: Physiological and behavioural responses of wandering albatross chicks (*Diomedea exulans*) to novel and non-novel predators

**DOI:** 10.1007/s00359-026-01805-5

**Published:** 2026-04-14

**Authors:** Anais Cotton, Christophe Barbraud, Sarah Leclaire, Karine Delord, Aymeric Bodin, Antoine Stier, Cécile Ribout, Charline Parenteau, Jean-Baptiste Ferdy, Charlotte Bourgoin, Joël White, Frédéric Angelier, Pierrick Blanchard

**Affiliations:** 1 Centre de Recherche sur la Biodiversité et l’Environnement (CRBE), UMR 5300, Université de Toulouse, Toulouse INP, CNRS, IRD, Toulouse, France; 2 Centre d’Études Biologiques de Chizé, CNRS – La Rochelle Université, UMR 7372, 79360 Villiers-en-Bois, France; 3 Réserve Naturelle Nationale des Terres Australes Françaises, TAAF, rue Gabriel Dejean, 97458 Saint-Pierre, France; 4 Université de Strasbourg, CNRS, IPHC UMR 7178, 67000 Strasbourg, France; 5 Department of Biology, University of Turku, Turku, Finland; 6 Ecole Nationale Supérieure de Formation de l’Enseignement Agricole (ENSFEA), 31326 Castanet-Tolosan, France

**Correction: Journal of Comparative Physiology A**

10.1007/s00359-026-01793-6

In the original version of the article, Fig. 4 was inadvertently duplicated. As a result, Fig. 3 displayed the same two graphs as Fig. 4. In the revised version of the article, this error has been rectified by replacing the incorrect version of Fig. 3 by the correct version. For completeness and transparency, both the incorrect version and the correct version of Fig. 3 are shown below.

Incorrect version:


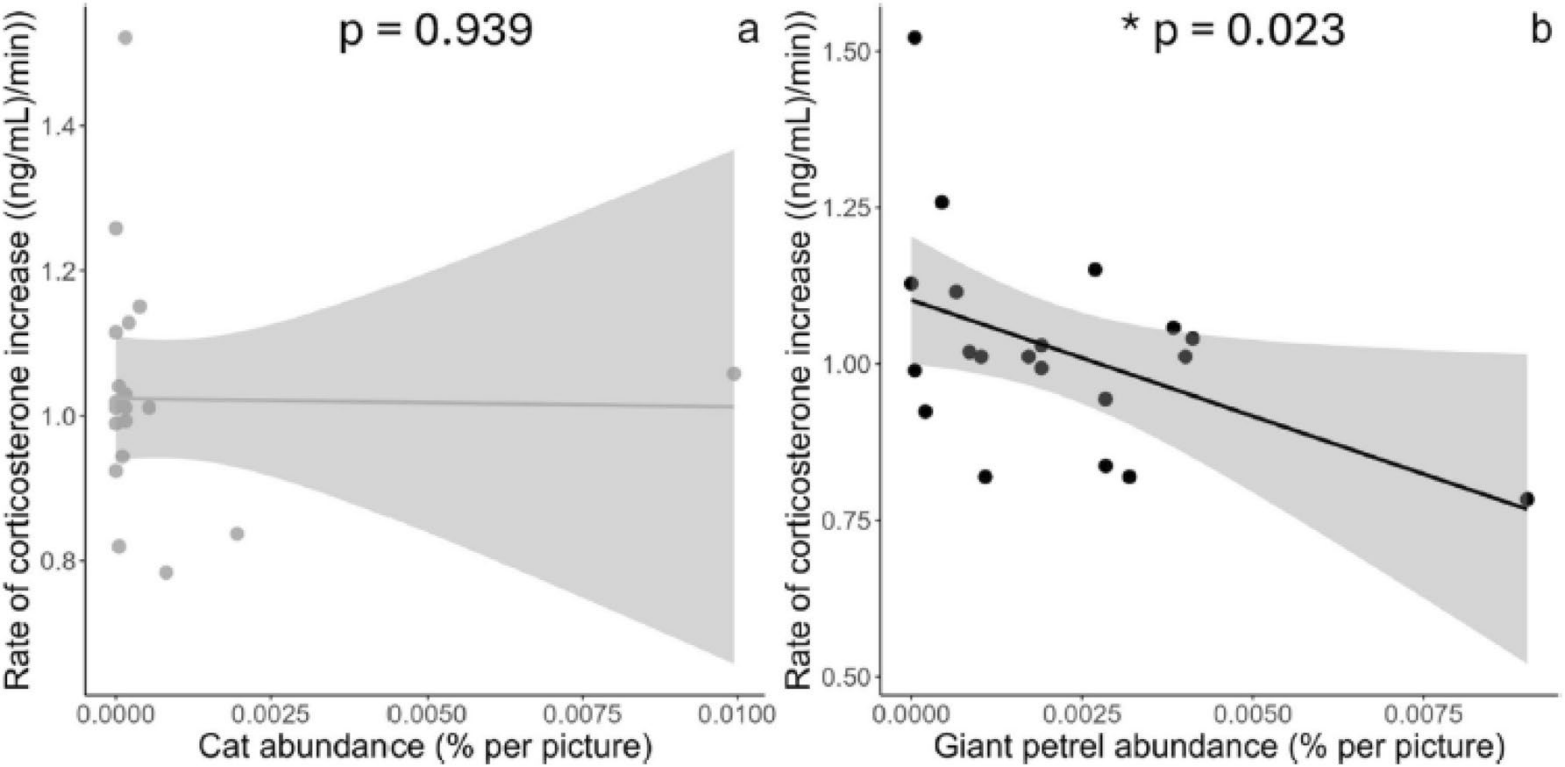


Correct version:


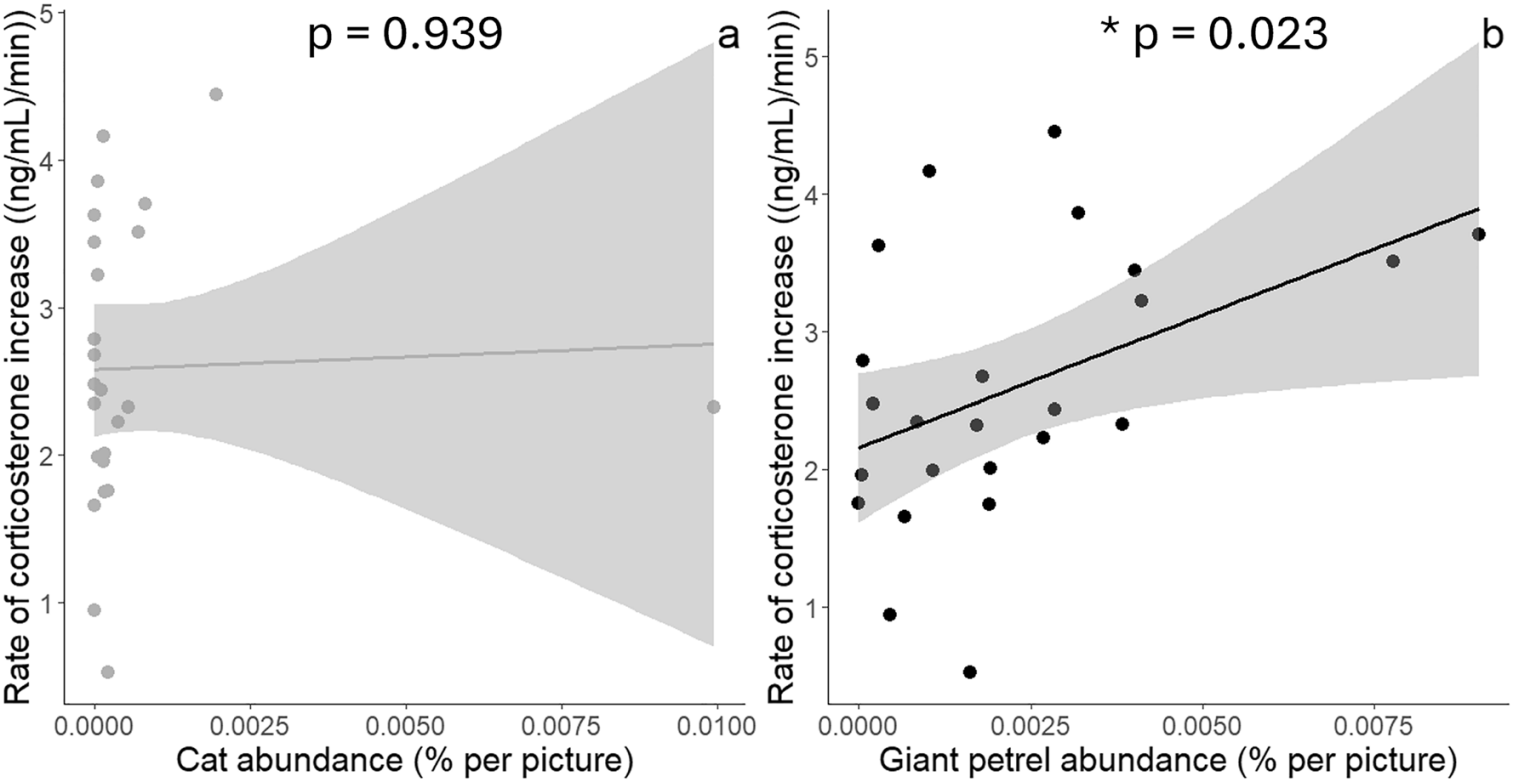


The original article has been corrected.

